# On the pedagogy of pharmacological communication: a study of final semester health science students

**DOI:** 10.1186/s12909-015-0467-2

**Published:** 2015-10-26

**Authors:** Ann Zetterqvist, Patrik Aronsson, Staffan Hägg, Karin Kjellgren, Margareta Reis, Gunnar Tobin, Shirley Booth

**Affiliations:** 1 Department of Pedagogical, Curricular and Professional Studies, University of Gothenburg, Gothenburg, Sweden; 2 Department of Pharmacology, Institute of Neuroscience and Physiology, The Sahlgrenska Academy, University of Gothenburg, Gothenburg, Sweden; 3 Department of Medical and Health Sciences, Division of Drug Research/Clinical Pharmacology, Linköping University, Linköping, Sweden; 4 School of Education, University of the Witwatersrand, Johannesburg, South Africa

**Keywords:** Pharmacology, Education, Communication, Phenomenography, Case description

## Abstract

**Background:**

There is a need to improve design in educational programmes for the health sciences in general and in pharmacology specifically. The objective of this study was to investigate and problematize pharmacological communication in educational programmes for the health sciences.

**Methods:**

An interview study was carried out where final semester students from programmes for the medical, nursing and specialist nursing in primary health care professions were asked to discuss the pharmacological aspects of two written case descriptions of the kind they would meet in their everyday work. The study focused on the communication they envisaged taking place on the concerns the patients were voicing, in terms of two features: how communication would take place and what would be the content of the communication. A phenomenographic research approach was used.

**Results:**

The results are presented as outcome spaces, sets of categories that describe the variation of ways in which the students voiced their understanding of communication in the two case descriptions and showed the qualitatively distinct ways in which the features of communication were experienced.

**Conclusions:**

The results offer a base of understanding the students’ perspectives on communication that they will take with them into their professional lives. We indicate that there is room for strengthening communication skills in the field of pharmacology, integrating them into programmes of education, by more widely implementing a problem-based, a case-oriented or role-playing pedagogy where final year students work across specialisations and there is a deliberate effort to evoke and assess advanced conceptions and skills.

## Background

Pharmacology as a part of the various programmes of health care education is of increasing importance for several reasons. The overall use of medications, multiple medications, and medicine-related problems is increasing in the general population [1]. Ever more drugs are being developed, not least to deal with the ills of an aging population [2]. Availability of both prescription and non-prescription drugs is increasing and today’s global mobility and Internet offer easy access to different types of drugs, from herbal remedies to drugs of abuse [3]. At the same time the amount of information accessible to the public and advertisements for drugs in different media is increasing. Communication between members of the health professions and the public needs to be considered in the light of these changes, and the professionals need to sharpen their communicative skills around pharmacological issues, or as we prefer to see it, develop an ability to draw on a depth of subject understanding to ensure patient understanding. In a study of the pharmacological knowledge of final semester students from three health science programmes (Aronsson et al, submitted) we have concluded that “participants were able to define pharmacological concepts but showed less ability to discuss the meaning of the concepts in depth”. Here we focus on communicating such pharmacological knowledge in clinical situations.

Across the broad spectrum of medical education, communication between professionals and patients is receiving increased attention; as Simmenroth-Nayda, Weiss, Fischer and Himmel [4] wrote, communication skills “are particularly important in primary care settings where diagnoses often may be obtained by an attentive history taking alone. Moreover, patient outcomes such as drug adherence, patient satisfaction and coping with illness depend, amongst other things, on the doctor’s communication abilities”. Diamantouror, Bartle and Geerts [5] add that for “provision of high-quality education improves compliance, increases time in the therapeutic range, and leads to a reduction in complications”. An extensive review of the literature [6] identifies three purposes of communication between physicians and their patients, namely creating a good interpersonal communication, exchanging information and making treatment-related decisions, and contrasts the vocabulary of the profession with everyday language. This complex view of what constitutes doctor-patient communication can be related even to other health care professions, with different factors being more or less important according to the professional role. There are calls to focus on communication throughout undergraduate education programmes and in-service training for medical professionals [7] – not only for future physicians but also for other health related professions such as dentistry [8] – and to study productive ways of enhancing communication skills by introducing different virtual patient settings [9].

The literature on education and communication between medical professionals and their patients has three distinct strands. First, there are curricular concerns in terms of the content of medical education and its assessment in an educational context [10] and methods for teaching communication skills [11]. Second, there are practical concerns in terms of evaluating programmes of communication education or communication skills acquired during medical training [12, 13]. And third, there is concern for communication less as a skill and more as an aspect of care for patients [14, 15]. Communicative competence is stressed by Skelton [16], drawing attention away from having skills towards deploying them appropriately, making the observation that communication is fundamentally a moral enterprise.

The study is grounded in an interest in improving the pedagogy of pharmacological communication in the health sciences. The aim was to investigate and problematize pharmacological communication in educational programmes for the health sciences as a first step. Specifically, the goal of the study is to chart the ways in which final semester students articulate their conceptions of the principal features of pharmacological communication in clinical cases – its nature and its content – to provide an element of student experience in the design and implementation of relevant educational programmes.

## Method

This study is part of a broader investigation on students’ understanding of pharmacological concepts (Aronsson et al, submitted) and their communication in professional practice.

The data consists of interviews with 12 final semester students from two Swedish universities (6 students each), from three different programmes, so that the participants include future doctors (4 students), future nurses (4 students) and experienced nurses who have trained to become specialist nurses in primary health care with the right to prescribe drugs (4 students). There was no intention to compare the performance of graduates from different programmes or from different universities, but the spread of backgrounds was intended to give a qualitative variation relevant for all of them. The study does not involve any handling of sensitive personal data or clinical procedures. All interviewees gave their informed consent prior to their inclusion in the study. The project complies fully with current applicable Swedish legal rules and ethical guidelines including the Helsinki declaration.

The students were individually presented with two written cases describing patients treated with a combination of drugs. The cases were designed to imitate a common health care provider-patient interaction. The study participants did not have access to Internet and the only tool provided was a hardcopy of FASS, the Swedish *national drug formulary* that provides healthcare professionals with detailed information about approved pharmaceuticals. Each student was given 30 min (15 min per case) in which to identify the pharmacological problems and find possible solutions. Immediately thereafter, the student discussed the cases for approximately 30 min with an interviewer (MR).

### The two case descriptions

These two case descriptions were presented to the interviewees for discussion. [Note that the contents of the square brackets were not in the original case description; only brand names were given].

#### Case A

Erik, age 56, comes for a follow-up visit at the health care centre where you meet him. Erik had a myocardial infarction 5 years ago and since then has been treated with Plavix [clopidogrel, an inhibitor of platelet aggregation]. A year ago Erik was treated with Omeprazol [omeprazol; a proton pump inhibitor], prescribed for symptoms of gastritis. The problems decreased but during recent months they have reappeared with symptoms of epigastralgia and nausea. Due to this Erik has started the Omeprazol treatment again. He now reports in this follow-up visit that he has been taking this drug for the past few weeks.

#### Case B

You meet Gunvor, age 74, at the health care centre. She has been using Trombyl [acetylsalicylic acid; 75 mg] for many years as a “blood thinner” to prevent a myocardial infarction and stroke. She now wants Trombyl replaced by another drug. The reason she gives is that she has read that Trombyl and Magnecyl [acetylsalicylic acid; 500 mg] are the same drug [acetylsalicylic acid]. She often takes Magnecyl for headaches and claims to have noted that Magnecyl has become less effective for pain relief. Gunvor’s daughter has also read on the Internet that Trombyl has a long effect duration with regards to blood platelets and finds it strange that Magnecyl does not last as long for headache. Gunvor asks if she can’t take another pain killer such as Ipren [ibuprofen] or Alvedon [paracetamol/acetaminophen] as a “blood thinner” instead.

The interviews were semi-structured, designed and executed to capture as much of the students knowledge and competence as possible. The opening questions for a discussion on communication were:

- What is it important for Erik/Gunvor to understand?
- How would you communicate this to him/her?
- Might there be obstacles for Erik/Gunvor to understand your message?
- How would you deal with that?

The participants were asked these questions towards the end of their interviews, after discussing the cases from a purely pharmacological perspective. A paper submitted elsewhere focused on the students’ understanding (Aronsson et al, submitted) of the pharmacological concepts pharmacodynamics, pharmacokinetics and drug interactions.

### Theoretical framework

The study was conceptualised in a phenomenographic methodological framework, which is to say that the researchers were aiming to reveal the qualitative variation of ways in which others understood the phenomenon in focus. Stenfors-Hayes, Hult and Dahlgren [17] introduce the research approach for particular use in medical education; Fyrenius, Silén and Wirell [18] make use of it to understand students’ meaning-making in a PBL context; and Marton and Booth [19] develop a theory of learning grounded in empirical research results.

Phenomenography is an empirical educational research approach, where a phenomenon is focused on in terms of the variation of ways in which people experience (or conceptualise, see or think about) it; this is to take a second-order perspective on the phenomenon, in contrast to research from a first-order perspective where it is the phenomenon as it exists in the world that is in focus. As a simple example, from a second order perspective, many people conceptualise tooth cavities as being caused by eating unsuitable foods, or by failing to brush enough, while from a first order perspective it is bacteria that cause the dental decay. The data collected for phenomenographic research is most often in the form of semi-structured open-ended interviews in which the phenomenon is considered from different openings, such as those that underpin this study. Analysis is often conducted in groups of educational researchers and subject specialists, as the constellation of pharmacological and educational researchers responsible for this article. The results are presented as a set of categories, called the *outcome space* of the study, which describe the variation in terms of qualitatively distinct categories that are related to the phenomenon rather than categorizing the individuals who voiced them.

It is the variation in the meaning that is expressed across the whole set of interviews that is of interest, rather than differences between individuals, as an individual can contribute to more than one category. As already stated, we are not comparing graduates from different universities, categories of future medical professionals or studying communication in authentic settings; what we are doing is to demonstrate that there are qualitatively different ways of conceptualising such communication across the set of interviews. We wish to understand what distinguishes one category from another as well as what constitutes a more complete and complex conceptualisation.

## Results

Two main thematic aspects were identified as constituting the phenomenon of pharmacological communication:

- What is pharmacological communication with a patient understood to be?
- What is seen to be the pharmacological content of the communication?

In this section, these two thematic aspects of communication are used as an organisational framework and will be described in terms of the qualitative variations found – as phenomenographic outcome spaces – illustrated by extracts from the interviews. The interviewees are identified by the order in which they were interviewed.

### Thematic aspect 1: What is pharmacological communication with a patient understood to be?

The analysis gave four qualitatively different ways of understanding the nature of pharmacological communication between the health care professional and the patient, varying basically between giving information, two-way communication on the medical professional’s premises, building communication on a hypothetical understanding of the patient and establishing the patient’s understanding from the outset.

#### Category 1. You give information, in the form of instruction and explanation

In this category the expression is of a *one-way communication* where the medical professional tells and the patient passively listens. One refers to the authority of FASS as a basis of information:

- S2: I would have, well, I’d have gone to FASS and that’s why you have to understand these different concepts so that I’d be able to explain. And then I’d have explained what I found there

Another student focuses on the potential consequences of Gunvor’s actions:

- S6: I’d explain as simply as possible in ordinary Swedish that it is pretty dangerous to take these medicines together, the risk of haemorrhage gets much higher

#### Category 2. You give information and check that the patient has understood

Now, the *patient also has a role* to play in potential communication after the consultation, as described here:

- S5: […] maybe I could ring her and ask how it’s going later on […] and if it seems a bit shaky so maybe I’d have to meet her in person and if it seems she still doesn’t understand it all – but I don’t want her to go home with questions but I would really want her to understand it there and then, so I’d call her up quite soon after. Her daughter is mentioned here, but I don’t know how their relation is. Would she want me to talk to her daughter?

Still, the patient is expected to be able to repeat, or ask apparently relevant questions about, or refer to a relative about what they have been told.

#### Category 3. You build your information on a hypothetical understanding of the patient and their understanding

In this third category, the health care professional takes a step further and *contemplates* what the patient might already understand, or might be capable of understanding, as here:

- S6: Well, I don’t know much about her actually. I know she is 74 and that … but who she is as a person, is she fully aware, is she senile, can she absorb information?

There, outward signs of the patient’s potential for communication are considered, whereas in the next extract more general attitudes are touched upon.

- S5: It depends on what the patient thinks about medicine, I think. Something you meet quite often is “I don’t like to take medicine, I don’t like tablets, the fewer tablets the better”

In either case, such contemplation can inform the communication that follows.

#### Category 4. You investigate what the patient understands and take it as a starting point for giving your information

In this, the most complex category, the health care professional takes an inquiring approach and *establishes* the patient’s potential for communication before embarking on an explanation or discussion on the medication

- S5: Yes, it’s important to investigate and wonder why she has headaches so often that she needs to take Magnecyl. […] She seems to have got the idea that the Trombyl isn’t working and we need to get her on board that it actually is working even if it isn’t helping against the headaches. Because it seems it’s there she goes “if it isn’t helping with this then it’s not helping with that either”

and:

- S3: Well, first you can ask her what she thinks, how she believes it is working. What she has the medicine for and how she thinks it is working. So she can explain in her own words…

These four categories refer to a general competence for communicating pharmacological advice, a relation between the health care professional and the patient. But all communication has *content* – the focus of the conversation that ensues. In this study the content was the medication that Erik and Gunvor were taking and the consequences of changing circumstances; this is the second theme of the phenomenographic analysis.

### Thematic aspect 2. What is seen to be the content of the pharmacological communication?

Three qualitatively different ways of seeing the content of the communication have been derived from the data, stripped of the actual pharmacological focus, but again ordered in a hierarchy from the least to the most complex and complete. The content concerns first what the patient must do, followed by, second, a justification of that, and third, explanation of different aspects of the medication.

#### Category 1. Instructions for adherence

First, the content of the pharmacological communication is that the prescribed drugs must be taken as *instructed:*

- S1: I think I am quite strict, so I want her to understand that she isn’t allowed to combine them, for exactly as she has already understood, they are exactly the same ingredients but that one has a much higher dose than the other

#### Category 2. Justification of why the patient should act in a certain way

In this category, instructions for adherence are assumed and *justification* becomes the content of the communication:

- S4: I would simply ask him what he knows about his medication and why he is taking them, because I know that’s important for compliance…

#### Category 3. Explanation of the relation between medicine, symptoms and potential consequences

In this third category, adherence and justification are complemented by *explanation*:

- S6: I’d tell her that because she is taking blood thinners for her heart attack and stroke because I want her blood to flow more easily, then if we increase that effect with Magnecyl then there is the risk that if she gets a knock she’ll get bruises and will bleed.

Even S4, cited above, goes on to say:

- S4: And if he doesn’t know I’d explain for him and encourage him to take both [Plavix and Omeprazol] so that… I don’t want to scare him too much but he could run a certain risk for bleeding ulcers.

These three categories form the outcome space in relation to the second thematic aspect: “What is seen to be the content of the pharmacological communication?” and give a picture of increasing trust in the ability of the patient to understand the reasons for medication. Rarely does the content of communication, as expressed in the study, involve finding out what the patient already understands of the symptoms and the medication, and that has to be seen as a shortcoming across the participants in the study.

## Discussion

This study shows that the variation in ways of understanding communication can vary from one-way instruction on how to act, to a two-way discussion where the patients’ understanding is elicited prior to an explanation of the symptoms and the diagnosis that is to be treated. It needs to be followed up with a study that can examine relations between depth of understanding pharmacological processes and ability to communicate them to patients. While it is not a study of communication in authentic clinical settings, it does highlight the fact that even in authentic clinical settings there are limits to the ways in which we can expect health care professionals to act; a person cannot be expected to communicate in a way that they are unable to articulate in a deep interview.

### The structure of the outcome spaces

The categories of the first outcome space, “What is pharmacological communication with a patient understood to be?” clearly form a hierarchy of less complex and less satisfactory to more complex and more complete ways of conceptualising pharmacological communication. The categories do not simply become more complex or additive, but they rather grow in engagement with the patient to take account of his or her concerns. What can this tell us about communication practices in clinical settings? In general, the capability for communicating is delimited by the way in which communication is conceptualised. One can imagine that a health professional, as S3, who is able to respond: “Well, first you can ask her what she thinks, how she believes it is working” from the fourth category, with the intention of finding out what the patient thinks about her medication, is more likely to encourage dialogue and mutual understanding than one who is limited to the first category, who would turn to the authoritative catalogue, as S2 does, and try to explain what he or she finds there, “I would have, well, I’d have gone to FASS”.

The hierarchical relationship of the second outcome space, the different ways in which the participants express the content of pharmacological communication, is less clear than their expression of the ways in which communication should be conducted. Certainly, the third category, where the patient is introduced to an explanation and prognosis, is most satisfying for a patient who can grasp and is interested in the details. The first and second, justifying adherence as they do, are also essential for an understanding of the importance to follow the medication regimen prescribed. An ideal response would take up all three, telling the patient what she may and may not do with an explanation of why that is so, and the consequences of the medicine for the symptoms.

### Consequences for teaching and learning in pharmacology

Earlier work with a phenomenographic approach to revealing qualitative differences in how students conceptualise phenomena of importance to their studies have made use of the variation they found in designing learning tasks [20]. It is necessary for a student to become aware that his or her taken-for-granted approach to communication could be otherwise. A physician, for instance, who sees her or his role as offering correct information (as in the first category) needs to understand that the information is not necessarily clear to the patient, and that hypotheses or investigations are needed to establish good communication (as in later categories). A nurse, for instance, who thinks that the advice given to a patient should focus on adherence to instruction (as in the first category) needs to become aware that in order to ensure adherence, the patient needs to be offered the opportunity to relate symptoms and diagnoses to the necessary medication (as in the third category).

Learning tasks, formulated as case studies or problems or role-playing exercises in courses in pharmacology, can be designed to open such variation of communicative approaches to students and thereby lead to discussions of what constitutes effective communication. Further, it enables assessment procedures to be based on an understanding of the complexity and completeness of approaches and content.

The results of the study can be compared with those to be found in the literature, revealed in ways other than that of our empirical research study. According to Moore, Wilkinson and Mercado [21] “research suggests that communication skills do not reliably improve with experience alone”, and although this is said in relation to communication around medical and psychological issues of cancer patients, it still puts considerable responsibility for ensuring that the basis of communication skills is laid down in higher education. One generally acknowledged tool for evaluating communication skills is the Kalamazoo Consensus Statement (KCS). This identifies essential elements of physician-patient communication as establishing rapport, opening discussion, gathering information, understanding a patient’s perspective of illness, sharing information, reaching agreement on problems and plans, and providing closure [22, 23]. The criticism that can be levelled at such generalised evaluation tools is that they neglect the specific content of the communication, the knowledge that the medical professional wishes the patient to understand and act on, here pharmacological knowledge. Within the confines of the case descriptions provided for the interviews in this study, most of the KCS elements came out in discussion, and those were in the more complex and complete categories of the outcome spaces. Hence, we suggest that these elements should be central considerations when designing education for communication skills and for evaluating the results.

Our analysis of the pharmacological knowledge of final semester students (Aronsson et al, submitted) indicates clearly that a thorough knowledge of the fundaments of pharmacology is essential for adequate communication. However, a good knowledge of the field does not necessarily ensure a good communication skill, as our study participants sometimes expressed when asked about obstacles to communication. A future research study could build on the results of these two studies to consider links between content knowledge and communication approach across a wider range of final semester students.

As we have already pointed out indirectly, the study has certain limitations. First, it is based on students’ discussions rather than on their actions, but this has enabled us to explore and chart the conceptions of communication held by final semester students across health sciences; it could act as grounding for a more observational study. Secondly, the number of interviewed students is relatively small, though it does cover a wide range of backgrounds; it could, however, act as a pilot for a questionnaire-based study of a larger number of graduating students with the possibility of correlating communication skills with knowledge of pharmacology. Thirdly, the implications for teaching practice are only outlined; it could, however, act as a framework for the design and evaluation of the elements of health science educational programmes that build communication skills.

A course in communication, we can conclude, must not simply focus on handling well-understood information in a stylised manner; pharmacological communication needs to be integrated into subject matter courses and practical work. Simmenroth-Nayda et al. [4] also conclude “[Communication skills] should be taught more in a problem-based method (“experimental”) than with instructional teaching methods”. We can add here that role playing and case-based studies offer similar opportunities for students at the end of their studies to be exposed to the issues of pharmacological communication across their specialisations.

## Conclusion

In this paper we have presented a study of understanding pharmacological communication in terms of the variation in ways in which final semester students of the health professions give expression to their potential for communication with patients in two different commonly occurring situations. The picture we have presented is not quantitative in nature, but gives a more nuanced qualitative description of what constitutes the understanding of communication. Further, we have suggested how it can provide an input into improving skills in pharmacological communication in the health science programmes.

In summary we have shown that:

- There is a qualitative variation in how final semester health care students approach issues of communicating pharmacological information, that has implications for the outcome of the communication.
- Health science students need to develop an ability to draw on a depth of understanding of pharmacological processes to ensure patient understanding and, thence, adherence.
- Communication skills needs to be considered as an essential and integrated aspect of health science education

Our last word is taken from the words of Henning Mankell, the internationally known Swedish author, who wrote of his treatment for cancer in The Guardian [24]. “But the doctors I remember most clearly are those who have displayed what can be described, certainly by me, as the innermost subtleties of the art of medical treatment. Alleviation, consolation, perhaps even cure, always involves a dialogue in which the patient and doctor learn how to talk to one another, and if possible create continuity. Medication and other treatments are never enough in themselves. If the patient doesn't understand what the doctor is saying, or if the doctor is unwilling or unable to interpret the questions and worries of the patient, the dialogue that is at the very heart of medical treatment will never materialise.” This places our pedagogical concern in a wider context of the patients and what they experience and appreciate in “the dialogue that is at the very heart of medical treatment”.
